# 4,4′-Bipyridine–5-fluoro­isophthalic acid (1/1)

**DOI:** 10.1107/S1600536811004016

**Published:** 2011-02-09

**Authors:** Jin-Lai Qin

**Affiliations:** a SINOPEC Catalyst Company, Beijing 100011, People’s Republic of China

## Abstract

Co-crystallization of 5-fluoro­isophthalic acid (H_2_fip) with 4,4′-bipyridine (bipy) leads to the formation of the title compound [(H_2_fip)(bipy)], C_8_H_5_FO_4_·C_10_H_8_N_2_, with an acid–base molar ratio of 1:1. The acid and base subunits are arrange alternately in the crystal structure, displaying a wave-like tape motif *via* inter­molecular O—H⋯N and C—H⋯O hydrogen bonds [carbox­yl–pyridine synthon of *R*_2_^2^(7) hydrogen-bond notation], which are further combined into a two-dimensional architecture through C—H⋯F inter­actions involving the bipy and H_2_fip mol­ecules.

## Related literature

For the supra­molecular synthon approach in crystal engineering, see: Desiraju (1995 ▶); Nangia & Desiraju (1998 ▶). For background to co-crystallization, see: Aakeröy & Salmon (2005 ▶); Sharma & Zaworotko (1996 ▶); Schultheiss & Newman (2009 ▶). For co-crystals with a carbox­yl–pyridyl heterosynthon, see: Etter (1990 ▶); Shan *et al.* (2002 ▶); Du *et al.* (2005 ▶). For co-crystals of halogen-substituted dicarb­oxy­lic acids, see: He *et al.* (2009 ▶).
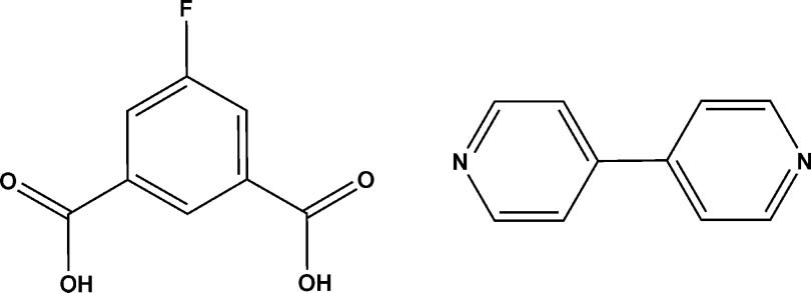


## Experimental

### 

#### Crystal data

C_8_H_5_FO_4_·C_10_H_8_N_2_
*M* *_r_* = 340.30
Monoclinic, 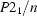

*a* = 7.1711 (13) Å
*b* = 20.106 (4) Å
*c* = 11.272 (2) Å
β = 106.781 (2)°
*V* = 1556.0 (5) Å^3^
*Z* = 4
Mo *K*α radiation
μ = 0.11 mm^−1^
*T* = 296 K
0.34 × 0.32 × 0.32 mm

#### Data collection

Bruker APEXII CCD area-detector diffractometer
Absorption correction: multi-scan (*SADABS*; Sheldrick, 2003 ▶) *T*_min_ = 0.963, *T*_max_ = 0.967
11109 measured reflections
2742 independent reflections
1956 reflections with *I* > 2σ(*I*)
*R*_int_ = 0.042

#### Refinement

*R*[*F* ^2^ > 2σ(*F* ^2^)] = 0.059
*wR*(*F* ^2^) = 0.166
*S* = 1.09
2742 reflections
228 parameters
1 restraint
H-atom parameters constrained
Δρ_max_ = 0.72 e Å^−3^
Δρ_min_ = −0.33 e Å^−3^

### 

Data collection: *APEX2* (Bruker, 2007 ▶); cell refinement: *APEX2* and *SAINT* (Bruker, 2007 ▶); data reduction: *SAINT*; program(s) used to solve structure: *SHELXTL* (Sheldrick, 2008 ▶); program(s) used to refine structure: *SHELXTL*; molecular graphics: *SHELXTL* and *DIAMOND* (Brandenburg, 2005 ▶); software used to prepare material for publication: *SHELXTL*.

## Supplementary Material

Crystal structure: contains datablocks I, global. DOI: 10.1107/S1600536811004016/vm2069sup1.cif

Structure factors: contains datablocks I. DOI: 10.1107/S1600536811004016/vm2069Isup2.hkl

Additional supplementary materials:  crystallographic information; 3D view; checkCIF report

## Figures and Tables

**Table 1:** Hydrogen-bond geometry (Å, °)

*D*—H⋯*A*	*D*—H	H⋯*A*	*D*⋯*A*	*D*—H⋯*A*
O1—H1*A*⋯N2	0.82	1.86	2.684 (3)	179
O3—H3⋯N1^i^	0.82	1.88	2.674 (4)	164
C8—H8⋯O2	0.93	2.42	3.138 (4)	134
C8—H8⋯F1^ii^	0.93	2.48	3.101 (4)	125

Symmetry codes: (i) 
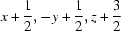
; (ii) 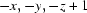.

**Fig. 1.**
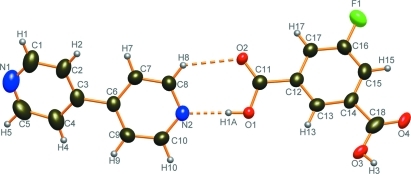
The molecular structure of compound (I) drawn with 30% probability ellipsoids.

**Fig. 2.**
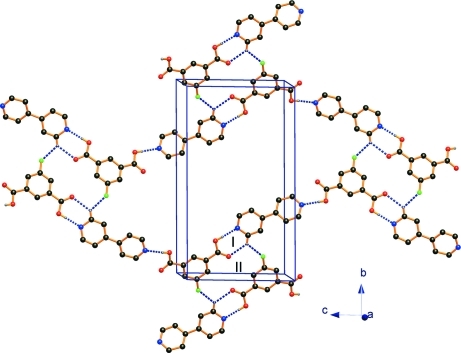
Two-dimensional hydrogen-bonded layer of (I). Hydrogen bonds are indicated as dashed lines. I and II indicate the synthons R22(7) and R24(14), respectively.

## supplementary crystallographic information

### Comment

In light of the importance of hydrogen bonds in crystal engineering, the supramolecular synthon approach has been widely applied to adapt desired supramolecules by using identified robust intermolecular interactions (Desiraju, 1995; Nangia & Desiraju, 1998). Co-crystallization is a current theme in several research groups to study hydrogen bonding through X-ray diffraction technique (Aakeröy & Salmon, 2005), for the synthesis of interpenetrated networks (Sharma & Zaworotko, 1996), and especially in pharmaceutical developments (Schultheiss & Newman, 2009). At this stage, strong hydrogen bonds, such as O—H···N or charge-assisted N—H···O, are always essential in the co-crystallization of carboxylic acids with pyridyl bases, usually combining the auxiliary weak C—H···O interactions, lead to the familiar carboxyl/pyridyl heterosynthon [*R*_2_^2^(7)] (Shan *et al.*, 2002). Although aromatic dicarboxylic acids have been verified to be excellent building blocks in binary co-crystal assemblies with bipyridine-type components (Du *et al.*, 2005), halogen substituented dicarboxylic acids have been seldom studied in this aspect (He, *et al.*, 2009). Doubtless, substituents will profoundly influence the structural assemblies by demonstrating distinct hydrogen-bonding capability and potential steric/electronic effect. To further investigate the hydrogen-bonding networks involving halogen substituents, 5-fluoroisophthalic acid (H_2_fip) was chosen to construct binary cocrystal with familiar 4,4'-bipyridine (bipy) component as a hydrogen-bonding participant for the first time.

In this work, the reaction of 5-fluoroisophthalic acid (H_2_fip) with 4,4'-bipyridine (bipy) under ambient conditions and evaporation from the mixed CH_3_OH/H_2_O (2:1) solution of the reactants yields the crystalline binary adduct [(H_2_fip)(bipy)] (I). Single crystal X-ray diffraction reveals that compound (I) contains one-dimensional supramolecular tape via the connection of predictable carboxylate-bipyridine O—H···N/C—H···O interactions of *R*_2_^2^(7) heterosynthon. Then, further C—H···F interactions extend the adjacent tape moieties into a two-dimensional (2-D) corrugated layer. The molecular structure contains one H_2_fip and one bipy molecule (Fig. 1). The two pyridyl rings within the basic unit form a dihedral angle of 30.9 (2) °. The heterosynthon *R*_2_^2^(7) ring pattern of O—H···N/C—H···O bonds (synthon I in Fig. 2, Table 1), connecting the base and acid moieties, is responsible for the formation of a 1-D wavelike tape structure. Analysis of the crystal packing of (I) suggests that a further C—H···F interaction (Table 1) expands the 1-D motif into a 2-D hydrogen-bonding network (Figu. 2). Within the 2-D layer, a new hydrogen-bonding pattern denoted as *R*_2_^4^(14) (synthon II in Fig. 2, Etter, 1990) is found to link two pairs of centrosymmetry related carboxyl-bipyridine motifs from adjacent tape structures. By comparison, a closely related 1:1 binary cocrystal of isophthalic acid and bipy exhibits similar tapes of acid:base components formed via *R*_2_^2^(7) synthons. But these tapes extend to form supramolecular sheets via additional C—H···O interactions (Shan *et al.*, 2002).

In conclusion, this work demonstrates the first example for H_2_fip as a good participant in co-crystallization with basic modules. When co-crystallizing with rod-like 4,4'-bipyridine building block, the H_2_fip subunits fulfill the reliable carboxylic-pyridine synthon *R*_2_^2^(7). Although the associated C—H···O bonds are not present between adjoining tape motifs, the introduction of fluorine substituents leads to a new hydrogen-bonding synthon *R*_2_^4^(14). This result presents a new challenge in the exploration of crystalline products based on such halogen substituted benzene dicarboxylic acids.

### Experimental

All the reagents and solvents for synthesis were commercially available and used as received. For the preparation of compound (I), to a CH_3_OH/H_2_O (2:1) solution (6 ml) of H_2_fip (18.4 mg, 0.1 mmol) was added a solution of bipy (15.8 mg, 0.1 mmol) in CH_3_OH (5 ml). After stirring for *ca.* 30 minutes, the reaction mixture was filtered and left to stand at ambient temperature. Colorless block crystals of (I) suitable for X-ray diffraction were gained through one week evaporation of the filtrate with a yield of 75 % (25.5 mg, based on bipy). Anal. Calcd for C_18_H_13_FN_2_O_4_: C, 63.53; H, 3.85; N, 8.23 %. Found: C, 63.50; H, 3.85; N, 8.29 %.

### Refinement

One restraint was applied to bonded N1 and C5 atoms to equalize each anisotropic vector component parallel to the bond (DELU command). H atoms bonded to C atoms were positioned geometrically (C—H = 0.93 Å for pyridyl and phenyl H atoms) and included in the refinement in the riding-model approximation, with *U*_iso_(H) = 1.2 *U*_eq_(C). O-bound H atoms were refined as rigid groups, allowed to rotate but not tip. Isotropic displacement parameters were derived from the parent atoms with *U*_iso_(H) = 1.5 *U*_eq_(O) and O—H distance of 0.82 Å.

### Crystal data

**Table:**

C_8_H_5_FO_4_·C_10_H_8_N_2_	*F*(000) = 704
*M**_r_* = 340.30	*D*_x_ = 1.453 Mg m^−^^3^
Monoclinic, *P*2_1_/*n*	Mo *K*α radiation, λ = 0.71073 Å
Hall symbol: -P 2yn	Cell parameters from 2260 reflections
*a* = 7.1711 (13) Å	θ = 2.1–21.8°
*b* = 20.106 (4) Å	µ = 0.11 mm^−^^1^
*c* = 11.272 (2) Å	*T* = 296 K
β = 106.781 (2)°	Block, colorless
*V* = 1556.0 (5) Å^3^	0.34 × 0.32 × 0.32 mm
*Z* = 4	

### Data collection

**Table:**

Bruker APEXII CCD area-detector diffractometer	2742 independent reflections
Radiation source: fine-focus sealed tube	1956 reflections with *I* > 2σ(*I*)
graphite	*R*_int_ = 0.042
φ and ω scans	θ_max_ = 25.0°, θ_min_ = 2.0°
Absorption correction: multi-scan (*SADABS*; Sheldrick, 2003)	*h* = −8→8
*T*_min_ = 0.963, *T*_max_ = 0.967	*k* = −23→22
11109 measured reflections	*l* = −13→13

### Refinement

**Table:**

Refinement on *F*^2^	Primary atom site location: structure-invariant direct methods
Least-squares matrix: full	Secondary atom site location: difference Fourier map
*R*[*F*^2^ > 2σ(*F*^2^)] = 0.059	Hydrogen site location: inferred from neighbouring sites
*wR*(*F*^2^) = 0.166	H-atom parameters constrained
*S* = 1.09	*w* = 1/[σ^2^(*F*_o_^2^) + (0.067*P*)^2^ + 1.097*P*] where *P* = (*F*_o_^2^ + 2*F*_c_^2^)/3
2742 reflections	(Δ/σ)_max_ < 0.001
228 parameters	Δρ_max_ = 0.72 e Å^−^^3^
1 restraint	Δρ_min_ = −0.33 e Å^−^^3^

### Special details

**Table:**

Geometry. All esds (except the esd in the dihedral angle between two l.s. planes) are estimated using the full covariance matrix. The cell esds are taken into account individually in the estimation of esds in distances, angles and torsion angles; correlations between esds in cell parameters are only used when they are defined by crystal symmetry. An approximate (isotropic) treatment of cell esds is used for estimating esds involving l.s. planes.
Refinement. Refinement of F^2^ against ALL reflections. The weighted R-factor wR and goodness of fit S are based on F^2^, conventional R-factors R are based on F, with F set to zero for negative F^2^. The threshold expression of F^2^ > 2sigma(F^2^) is used only for calculating R-factors(gt) etc. and is not relevant to the choice of reflections for refinement. R-factors based on F^2^ are statistically about twice as large as those based on F, and R- factors based on ALL data will be even larger.

### Fractional atomic coordinates and isotropic or equivalent isotropic displacement parameters (Å^2^)

**Table:**

	*x*	*y*	*z*	*U*_iso_*/*U*_eq_	
C1	0.2245 (5)	0.3051 (2)	−0.1085 (3)	0.0680 (10)	
H1	0.2529	0.2771	−0.1667	0.082*	
C2	0.2595 (5)	0.28299 (18)	0.0108 (3)	0.0578 (9)	
H2	0.3103	0.2406	0.0319	0.069*	
C3	0.2198 (4)	0.32356 (15)	0.1013 (3)	0.0463 (7)	
C4	0.1530 (5)	0.38653 (18)	0.0671 (3)	0.0618 (9)	
H4	0.1293	0.4163	0.1242	0.074*	
C5	0.1208 (5)	0.40493 (18)	−0.0617 (4)	0.0681 (9)	
H5	0.0760	0.4475	−0.0870	0.082*	
C6	0.2374 (4)	0.29661 (14)	0.2275 (2)	0.0390 (6)	
C7	0.2065 (4)	0.22960 (14)	0.2432 (3)	0.0472 (7)	
H7	0.1809	0.2009	0.1758	0.057*	
C8	0.2138 (5)	0.20553 (15)	0.3592 (3)	0.0484 (7)	
H8	0.1927	0.1603	0.3672	0.058*	
C9	0.2766 (5)	0.33605 (15)	0.3328 (3)	0.0512 (8)	
H9	0.2999	0.3813	0.3279	0.061*	
C10	0.2807 (5)	0.30759 (15)	0.4452 (3)	0.0522 (8)	
H10	0.3070	0.3350	0.5146	0.063*	
C11	0.1900 (4)	0.11407 (14)	0.6366 (3)	0.0420 (7)	
C12	0.1879 (4)	0.07233 (13)	0.7462 (2)	0.0374 (6)	
C13	0.2825 (4)	0.09158 (14)	0.8674 (2)	0.0401 (7)	
H13	0.3453	0.1325	0.8829	0.048*	
C14	0.2827 (4)	0.04932 (14)	0.9653 (2)	0.0431 (7)	
C15	0.1878 (5)	−0.01089 (15)	0.9423 (3)	0.0498 (8)	
H15	0.1868	−0.0393	1.0072	0.060*	
C16	0.0951 (5)	−0.02820 (14)	0.8225 (3)	0.0482 (7)	
C17	0.0933 (4)	0.01109 (14)	0.7225 (3)	0.0424 (7)	
H17	0.0313	−0.0027	0.6420	0.051*	
C18	0.3876 (5)	0.06363 (18)	1.0980 (3)	0.0558 (9)	
F1	0.0045 (3)	−0.08851 (9)	0.80111 (18)	0.0751 (7)	
N1	0.1528 (4)	0.36369 (18)	−0.1434 (3)	0.0707 (8)	
N2	0.2493 (4)	0.24332 (12)	0.4599 (2)	0.0453 (6)	
O1	0.2483 (3)	0.17584 (10)	0.66511 (18)	0.0521 (6)	
H1A	0.2473	0.1961	0.6018	0.078*	
O2	0.1424 (4)	0.09213 (11)	0.53240 (18)	0.0615 (7)	
O3	0.4816 (4)	0.12082 (13)	1.11386 (19)	0.0662 (7)	
H3	0.5535	0.1231	1.1850	0.099*	
O4	0.3880 (4)	0.02582 (13)	1.1812 (2)	0.0745 (8)	

### Atomic displacement parameters (Å^2^)

**Table:**

	*U*^11^	*U*^22^	*U*^33^	*U*^12^	*U*^13^	*U*^23^
C1	0.065 (2)	0.091 (3)	0.046 (2)	−0.003 (2)	0.0142 (17)	0.0128 (19)
C2	0.061 (2)	0.075 (2)	0.0364 (17)	−0.0063 (17)	0.0124 (15)	0.0050 (16)
C3	0.0388 (15)	0.0540 (18)	0.0413 (17)	−0.0042 (13)	0.0037 (13)	0.0110 (14)
C4	0.062 (2)	0.060 (2)	0.061 (2)	0.0019 (16)	0.0123 (17)	0.0205 (17)
C5	0.068 (2)	0.052 (2)	0.078 (2)	0.0076 (17)	0.0099 (19)	0.0185 (15)
C6	0.0388 (15)	0.0420 (16)	0.0348 (15)	−0.0001 (12)	0.0081 (12)	0.0051 (12)
C7	0.0608 (19)	0.0430 (17)	0.0358 (16)	−0.0047 (14)	0.0110 (14)	−0.0032 (13)
C8	0.0643 (19)	0.0401 (17)	0.0395 (17)	−0.0050 (14)	0.0130 (14)	0.0029 (13)
C9	0.072 (2)	0.0363 (16)	0.0461 (18)	−0.0036 (15)	0.0182 (16)	0.0005 (13)
C10	0.074 (2)	0.0449 (18)	0.0371 (17)	−0.0070 (15)	0.0158 (15)	−0.0064 (13)
C11	0.0455 (16)	0.0424 (17)	0.0364 (16)	−0.0014 (13)	0.0090 (13)	−0.0012 (13)
C12	0.0407 (15)	0.0392 (15)	0.0317 (14)	0.0026 (12)	0.0095 (12)	0.0003 (11)
C13	0.0442 (16)	0.0368 (15)	0.0377 (15)	0.0009 (12)	0.0092 (12)	−0.0053 (12)
C14	0.0488 (16)	0.0508 (18)	0.0285 (14)	0.0109 (13)	0.0091 (12)	0.0004 (12)
C15	0.067 (2)	0.0463 (18)	0.0365 (16)	0.0066 (15)	0.0154 (15)	0.0074 (13)
C16	0.0629 (19)	0.0372 (16)	0.0444 (17)	−0.0057 (14)	0.0156 (15)	0.0032 (13)
C17	0.0491 (17)	0.0424 (16)	0.0325 (15)	−0.0023 (13)	0.0066 (13)	−0.0017 (12)
C18	0.059 (2)	0.067 (2)	0.0395 (18)	0.0141 (17)	0.0113 (15)	−0.0101 (17)
F1	0.1104 (17)	0.0482 (11)	0.0623 (12)	−0.0268 (11)	0.0179 (11)	0.0032 (9)
N1	0.0663 (19)	0.091 (2)	0.0517 (18)	−0.0038 (17)	0.0114 (14)	0.0132 (14)
N2	0.0574 (15)	0.0436 (14)	0.0347 (13)	−0.0042 (11)	0.0131 (11)	0.0021 (11)
O1	0.0731 (14)	0.0454 (12)	0.0361 (11)	−0.0108 (10)	0.0129 (10)	0.0004 (9)
O2	0.0936 (18)	0.0562 (14)	0.0324 (12)	−0.0184 (12)	0.0147 (11)	−0.0025 (10)
O3	0.0738 (16)	0.0816 (18)	0.0352 (12)	0.0014 (13)	0.0030 (11)	−0.0124 (11)
O4	0.101 (2)	0.0853 (18)	0.0302 (12)	0.0180 (15)	0.0084 (12)	0.0083 (12)

### Geometric parameters (Å, °)

**Table:**

C1—N1	1.301 (5)	C10—H10	0.9300
C1—C2	1.368 (4)	C11—O2	1.208 (3)
C1—H1	0.9300	C11—O1	1.320 (3)
C2—C3	1.398 (4)	C11—C12	1.497 (4)
C2—H2	0.9300	C12—C13	1.393 (4)
C3—C4	1.369 (4)	C12—C17	1.394 (4)
C3—C6	1.493 (4)	C13—C14	1.392 (4)
C4—C5	1.451 (5)	C13—H13	0.9300
C4—H4	0.9300	C14—C15	1.376 (4)
C5—N1	1.308 (5)	C14—C18	1.496 (4)
C5—H5	0.9300	C15—C16	1.367 (4)
C6—C7	1.385 (4)	C15—H15	0.9300
C6—C9	1.387 (4)	C16—F1	1.364 (3)
C7—C8	1.381 (4)	C16—C17	1.373 (4)
C7—H7	0.9300	C17—H17	0.9300
C8—N2	1.328 (4)	C18—O4	1.207 (4)
C8—H8	0.9300	C18—O3	1.319 (4)
C9—C10	1.382 (4)	O1—H1A	0.8200
C9—H9	0.9300	O3—H3	0.8200
C10—N2	1.330 (4)		
			
N1—C1—C2	122.4 (4)	C9—C10—H10	118.1
N1—C1—H1	118.8	O2—C11—O1	124.1 (3)
C2—C1—H1	118.8	O2—C11—C12	122.0 (3)
C1—C2—C3	120.6 (4)	O1—C11—C12	113.9 (2)
C1—C2—H2	119.7	C13—C12—C17	120.4 (2)
C3—C2—H2	119.7	C13—C12—C11	122.4 (2)
C4—C3—C2	117.6 (3)	C17—C12—C11	117.2 (2)
C4—C3—C6	122.0 (3)	C14—C13—C12	119.7 (3)
C2—C3—C6	120.3 (3)	C14—C13—H13	120.1
C3—C4—C5	117.3 (3)	C12—C13—H13	120.1
C3—C4—H4	121.3	C15—C14—C13	120.0 (3)
C5—C4—H4	121.3	C15—C14—C18	115.9 (3)
N1—C5—C4	122.3 (3)	C13—C14—C18	124.1 (3)
N1—C5—H5	118.9	C16—C15—C14	118.9 (3)
C4—C5—H5	118.9	C16—C15—H15	120.6
C7—C6—C9	116.6 (2)	C14—C15—H15	120.6
C7—C6—C3	120.1 (3)	F1—C16—C15	118.2 (3)
C9—C6—C3	123.3 (3)	F1—C16—C17	118.4 (3)
C8—C7—C6	119.9 (3)	C15—C16—C17	123.4 (3)
C8—C7—H7	120.1	C16—C17—C12	117.5 (3)
C6—C7—H7	120.1	C16—C17—H17	121.2
N2—C8—C7	123.7 (3)	C12—C17—H17	121.2
N2—C8—H8	118.2	O4—C18—O3	123.9 (3)
C7—C8—H8	118.2	O4—C18—C14	122.9 (3)
C10—C9—C6	119.5 (3)	O3—C18—C14	113.2 (3)
C10—C9—H9	120.2	C1—N1—C5	119.7 (3)
C6—C9—H9	120.2	C8—N2—C10	116.5 (2)
N2—C10—C9	123.8 (3)	C11—O1—H1A	109.5
N2—C10—H10	118.1	C18—O3—H3	109.5
			
N1—C1—C2—C3	−0.3 (5)	C17—C12—C13—C14	0.1 (4)
C1—C2—C3—C4	−2.7 (5)	C11—C12—C13—C14	−177.2 (2)
C1—C2—C3—C6	173.2 (3)	C12—C13—C14—C15	−0.7 (4)
C2—C3—C4—C5	2.7 (5)	C12—C13—C14—C18	176.9 (3)
C6—C3—C4—C5	−173.2 (3)	C13—C14—C15—C16	0.3 (4)
C3—C4—C5—N1	0.2 (5)	C18—C14—C15—C16	−177.6 (3)
C4—C3—C6—C7	146.8 (3)	C14—C15—C16—F1	178.9 (3)
C2—C3—C6—C7	−29.0 (4)	C14—C15—C16—C17	0.9 (5)
C4—C3—C6—C9	−30.5 (4)	F1—C16—C17—C12	−179.6 (3)
C2—C3—C6—C9	153.8 (3)	C15—C16—C17—C12	−1.5 (5)
C9—C6—C7—C8	0.6 (4)	C13—C12—C17—C16	1.0 (4)
C3—C6—C7—C8	−176.9 (3)	C11—C12—C17—C16	178.4 (3)
C6—C7—C8—N2	0.1 (5)	C15—C14—C18—O4	−1.2 (4)
C7—C6—C9—C10	−0.7 (4)	C13—C14—C18—O4	−179.0 (3)
C3—C6—C9—C10	176.7 (3)	C15—C14—C18—O3	177.7 (3)
C6—C9—C10—N2	0.1 (5)	C13—C14—C18—O3	−0.1 (4)
O2—C11—C12—C13	165.0 (3)	C2—C1—N1—C5	3.4 (5)
O1—C11—C12—C13	−14.8 (4)	C4—C5—N1—C1	−3.3 (5)
O2—C11—C12—C17	−12.3 (4)	C7—C8—N2—C10	−0.7 (5)
O1—C11—C12—C17	167.9 (2)	C9—C10—N2—C8	0.6 (5)

### Hydrogen-bond geometry (Å, °)

**Table:**

*D*—H···*A*	*D*—H	H···*A*	*D*···*A*	*D*—H···*A*
O1—H1A···N2	0.82	1.86	2.684 (3)	179
O3—H3···N1^i^	0.82	1.88	2.674 (4)	164
C8—H8···O2	0.93	2.42	3.138 (4)	134
C8—H8···F1^ii^	0.93	2.48	3.101 (4)	125

Symmetry codes: (i) *x*+1/2, −*y*+1/2, *z*+3/2; (ii) −*x*, −*y*, −*z*+1.
